# Rediscovery of *Achipteria setulosa*, with remarks on Japanese species of Achipteriidae and the proposal of species-groups (Acari, Oribatida)

**DOI:** 10.3897/zookeys.578.7603

**Published:** 2016-04-07

**Authors:** Ichiro Maruyama, Badamdorj Bayartogtokh, Satoshi Shimano

**Affiliations:** 1 640-3 Yokoyama, Kashiwazaki City, Niigata, 945-1101 Japan; 2 Department of Biology, School of Arts and Sciences, National University of Mongolia, Ulaanbaatar, 14201 Mongolia; 3 Science Research Center, Hosei University, Fujimi, Chiyoda-ku, Tokyo, 102-8160 Japan

**Keywords:** Achipteria, grassland on limestone, Izuachipteria, Japan, new record, species-group

## Abstract

The first detailed description of adults of *Achipteria setulosa* Golosova, 1981 with illustrations are provided, based on materials from central Japan. This species is placed in the subgenus Achipteria (Izuachipteria) Balogh & Mahunka, 1979. In addition, the species grouping of the known species in the genus *Achipteria* is briefly discussed, and three species-groups are proposed based on the structure of the lamellar complex. Furthermore, data on distribution, diversity and habitat ecology of all known species of Achipteriidae in Japan are presented, and a key is provided for the identification of recorded species in this country. The majority of achipteriid species found in Japan are known to be widely distributed in the vast areas of the northern hemisphere; only two species have restricted distributions in Japan. Most species of Achipteriidae in Japan are inhabitants of the litter of various forests, such as natural broad-leaved forests in high mountainous areas, soils of grasslands, wetlands and mosses growing on rocks.

## Introduction

The oribatid mites belonging to the family Achipteriidae Thor, 1929 occur frequently, even sometimes with high numbers, in forest soils, litters, meadow soils, liverworts, bogs and at edges of lakes with mosses, but rarely found in arboreal habitats. Representatives of this family are diverse in both northern and southern hemispheres, but in the tropics, achipteriid species are mainly found at high elevations, for example, in cloud forest litter. Achipteriid species whose feeding habits have been studied are saprophages and mycophages that apparently feed opportunistically on available resources of fungi, algae and decaying plant material (Root et al. 2007, Seniczak and Seniczak 2007, Lindo et al. 2008, Norton and Behan-Pelletier 2009).

Some species of Achipteriidae are sensitive to environmental changes, including pollutants, and therefore, they may indicate changes in habitats. Several species of this family serve as intermediate hosts of tapeworms of the superfamily Anoplocephalata, which parasitize on wild and domestic animals (Rajski 1959, Denegri 1993, Seniczak and Seniczak 2007). The family is known from the Holarctic, Oriental and Neotropical regions with most species described from the North America, Europe, Central America and East Asia.

Currently, the family Achipteriidae Thor, 1929 includes seven genera, three subgenera, 90 species and four subspecies (Subías 2004, 2015). Among the genera, *Achipteria* Berlese, 1885 is largest in terms of species richness, and it includes two subgenera and 35 species (including two subspecies). Most known species belong to the nomino-typical subgenus *Achipteria* (31 species, two subspecies).

Balogh and Mahunka (1979) proposed *Izuachipteria* and *Hokkachipteria* as new genera based on the character states of interlamellar setae, but Subías (2004) considered these as a subgenus of *Achipteria*. The main difference between subgenera *Achipteria* and *Izuachipteria* is size of interlamellar setae, which are long and thick, extending beyond basis of lamellar cusps in Achipteria (Achipteria), in contrast very short and slender interlamellar setae (or it is completely absent) in Achipteria (Izuachipteria). Only two species have hitherto been grouped into Achipteria (Izuachipteria), namely Achipteria (Izuachipteria) imperfecta (Suzuki, 1972) and Achipteria (Izuachipteria) alpestris (Aoki, 1973).

Eleven species of Achipteriidae have been recorded previously from Japan (Aoki 1959, 1961, 1970, 1973, 1976, Suzuki 1972, Fujikawa et al. 1993, Hirauchi and Aoki 1997, Maruyama 2003, Ohkubo et al. 2015).

The aim of the present work is to redescribe the morphology of a little known species, *Achipteria setulosa* Golosova, 1981, which is found for the first time in Japan. This species has character states of the subgenus Achipteria (Izuachipteria), therefore, we combine this species in the latter subgenus. Proposing the species grouping of the known species of *Achipteria* *sensu lato* along with review of the composition of the family Achipteriidae in Japan, with remarks on their biogeography, habitat ecology, and construction of an identification key to all known species from this country are the other goal of this study.

## Material and methods

In total 64 specimens (26 males and 38 females) were collected from litter and soil of the grassland with Saxifraga fortunei Hook. f. var. alpina Nakai in the bottom of Senridou Doline, Maikomi-Daira (limestone area), Itoigawa City, Niigata Prefecture, Japan, 36°57'37"N, 137°48'10"E, alt. 695 m a.s.l., 03 September 2007, collected by. I. Maruyama.

The morphological terminology used below is mostly that developed over many years by Grandjean (1932, 1952), and also that by Norton (1977), Norton and Behan-Pelletier (2009). The specimens were cleared in lactic acid and mounted on temporary slides to view the anterior, lateral and posterior aspects and then preserved in alcohol. A differential interference contrast microscope (Olympus BH 2) was used for investigation in transmitted light. Line drawings were made using a camera lucida attached to the compound microscope.

All measurements are given as a range, with the mean in parentheses. Body length was measured in lateral view, from the tip of the rostrum to the posterior edge of the ventral plate, to avoid discrepancies caused by different degrees of notogastral distension. Notogastral length was also measured in lateral aspect (when the dorsosejugal groove is discernable), from the anterior to the posterior edge; notogastral width refers to the maximum width in dorsal aspect. Setal formulas of the legs (including famulus) are given as numbers per segment for appendages (from trochanter to tarsus) and formulas of solenidia are given separately as number per podosomal segment.

## Description

### Achipteria (Izuachipteria) setulosa

Taxon classificationAnimaliaSarcoptiformesAchipteriidae

(Golosova, 1981) comb. n.

[Japanese name: Maikomi-tsunobanedani]

Figs 1 , 2

Achipteria setulosa Golosova, 1981: p. 148, fig. 1.
Achipteria setulosa : Pan’kov et al. 1997: p. 66; Bayartogtokh and Ryabinin 2012: p. 153.

#### Diagnosis.

Large species, body length: 718–796 μm; width: 480–576 μm (n = 10). Lamellar setae short, thin, smooth, inserted ventrally on cusps, not reaching tip of cusps; interlamellar setae short, thin, smooth, not reaching basis of lamellar cusps; sensilli long, club-shaped, epimeral regions III and IV with three setae each.

*Measurement*. Body length: 718–796 (759) μm; width: 480–576 (543) μm (n = 10).

*Integument*. Body color dark brown, heavily sclerotized species with minute microtubercles on lateral part of podosoma, exobothridial and lenticular regions. Granular cerotegument (with minute round to conical granular structure) clearly evident at base of prodorsum and on mentum.

**Figure 1.**
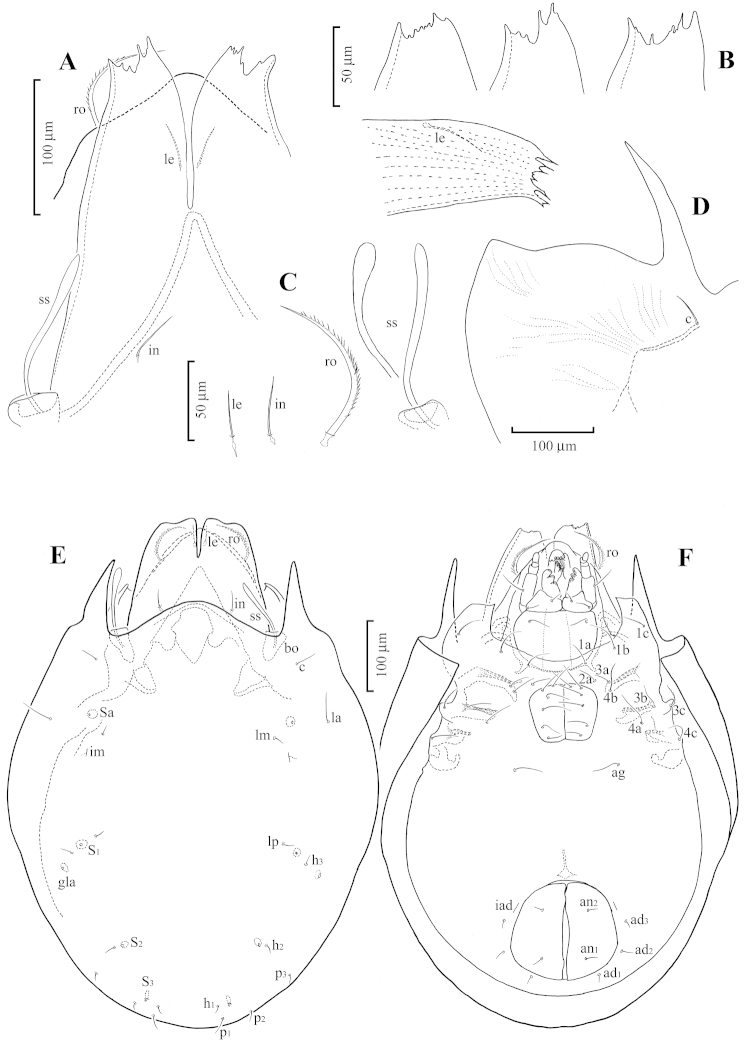
Achipteria (Izuachipteria) setulosa (Golosova, 1981). **A** Part of prodorsum showing flatly extended lamellae (after dissection) **B** Lamellar cusps, showing variation in arrangement of teeth on the anterior edge **C** Lamellar, interlamellar, rostral setae and sensillus showing variation of its head **D** Pteromorph showing its anterior projection and lateral corner (after dissection) **E** Dorsal view of body **F** Ventral view of body.

*Prodorsum* (Fig. 1A–C, E): Rostrum rounded, without horn-like anterior projection. Rostral setae (*ro*) long, barbed, curved inward, extending beyond tip of rostrum. Lamellae long and broad, fused medially; lamellar cusps nearly half as long as total length of lamellae, its anterior margin bending downwards, serrated irregularly as shown in Fig. 1A, B. Tutoria (*tu*) medium long, narrow, with free cusps distally. Lamellar setae (*le*) short (about 24 μm), thin, smooth, inserted ventrally on cusps, not exposed from cusps. Interlamellar setae (*in*) short, but slightly longer (about 35 μm) than lamellar setae, not reaching on base of lamellar cusps. Exobothridial setae not evident. Sensilli club-shaped, relatively long (about 102 μm), its head smooth (Fig. 1C). Bothridia nearly funnel-shaped, its opening exposed from anterior margin of notogaster.

*Notogaster* (Fig. 1D–F): Longer than wide, anterior and posterior margins broadly rounded. Lenticular region irregularly pentagonal, with diffuse margins, but weakly visible and lacking true lenticulus. Anterior projection of pteromorphs pointed, not reaching level of rostrum (Fig. 1D–F). Among 10 pairs of notogastral setae, *la* longest (35–42 μm), *c* next long setae (25–32 μm), other setae distinctly shorter (12–17 μm); relative length of mutual distances of setal pairs: *la*–*la* > *h*_3_–*h*_3_ > *c*–*c* > *p*_3_–*p*_3_ > *lp*–*lp* > *lm*–*lm* > *h*_2_–*h*_2_ > *p*_2_–*p*_2_ > *p*_1_–*p*_1_ > *h*_1_–*h*_1_. Four pairs of sacculi clearly developed; *Sa* located anterolaterally to setae *la*, *S*_1_ between setae *lp* and *h*_3_, *S*_2_ anteriomediad of setae *h*_2_, and *S*_3_ anterolaterally to setae *h*_1_. Lyrifssures *im* situated posterolaterally to setae *lm*. Openings of opisthonotal glands (*gla*) located posterolaterlly to setae *h*_3_.

*Gnathosoma* (Fig. 1F): Subcapitulum nearly as long as wide, smooth throughout; setae *h* 37 μm, *m* 17 μm, and *a* 15 μm, smooth. Chelicerae chelate-dentate (178 μm), cheliceral setae long, barbed, *cha* (64 μm) longer than *chb* (35 μm). Palps typical for family (104 μm), formula of setation: 0–2–1–3–10 including solenidion ω on tarsus.

*Epimeral and lateral podosomal regions* (Fig. 1F): Genal teeth rectangular, with pointed tip. Pedotecta I with pointed anteromedial end as seen in ventral view, and even more sharply pointed in lateral view. Apodemes *apo.2*, *apo.sj* and *apo.3* well developed. Epimeral regions III and IV with three setae each; epimeral setae 35–42 μm in length; *1c* and *3d* barbed, other setae smooth. Epimeral setal formula: 3–1–3–3. Custodia and discidia not clearly developed; circumpedal carinae poorly developed.

*Anogenital region* (Fig. 1F): Genital and aggenital setae long (36–43 μm), smooth; relative length of their mutual distances: *g*_5_–*g*_5_ > *g*_4_–*g*_4_ ≥ *g*_2_–*g*_2_ > *g*_3_–*g*_3_ > *g*_6_–*g*_6_ > *g*_1_–*g*_1_. Anal and adanal setae (13–18 μm) smooth; mutual distances of *an*_1_–*an*_1_ and *an*_2_–*an*_2_ almost equal; relative distances between anal and adanal setae: *ad*_1_–*ad*_1_ > *an*_1_–*an*_1_ > *an*_2_–*an*_2_ = *an*_1_–*an*_1_ > *ad*_1_–*ad*_1_ > *ad*_2_–*ad*_2_. Adanal lyrifissures (*iad*) aligned, almost parallel to anterolateral margins of anal aperture.

**Figure 2.**
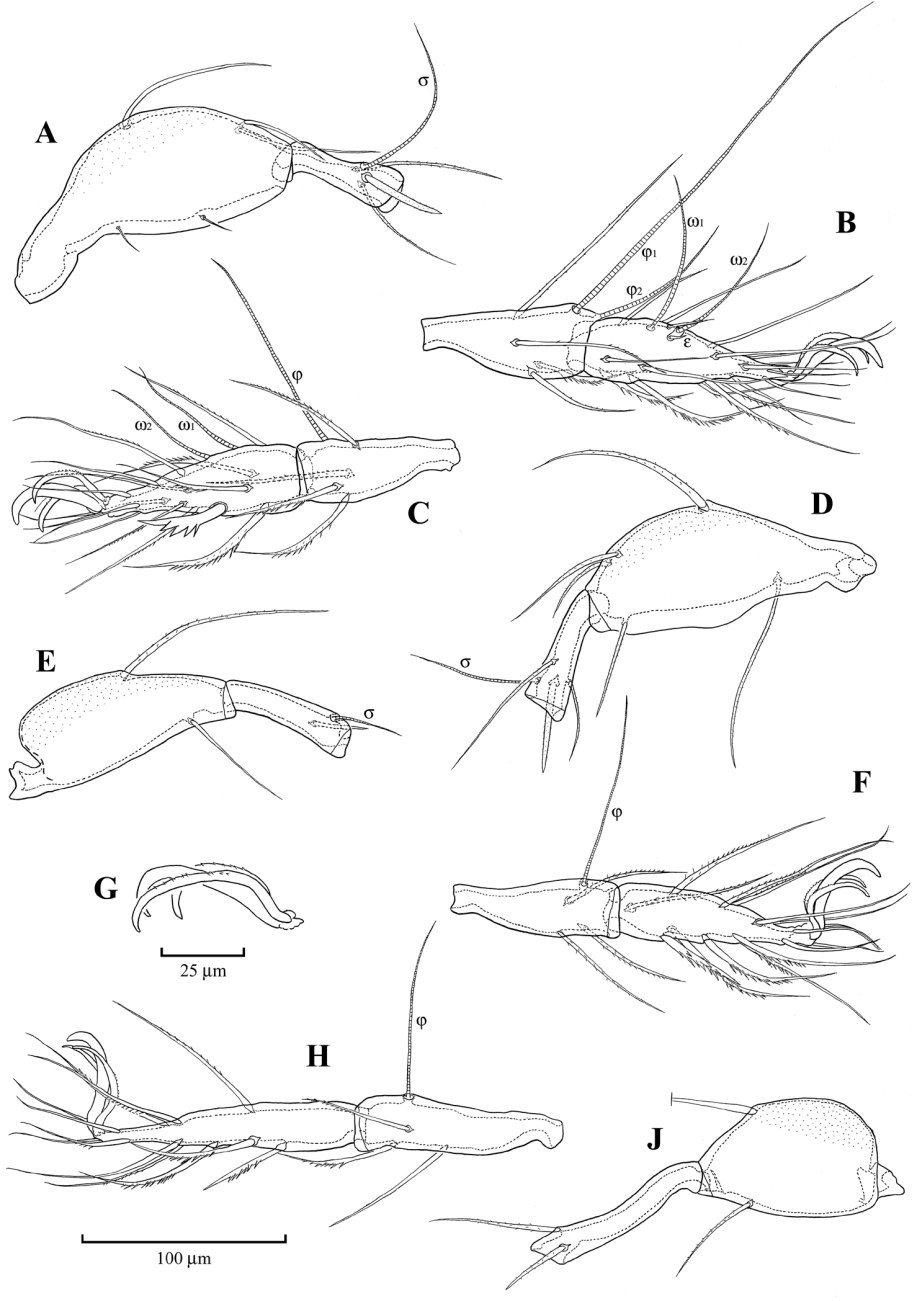
Achipteria (Izuachipteria) setulosa (Golosova, 1981). **A** Femur and genu of leg I (right, antiaxial aspect) **B** Tibia and tarsus of leg I (right, antiaxial aspect) **C** Tibia and tarsus of leg II (left, antiaxial aspect) **D** Femur and genu of leg II (left, antiaxial aspect) **E** Femur and genu of leg III (right, paraxial aspect) **F** Tibia and tarsus of leg III right, paraxial aspect) **G** Claws of leg IV **H** Tibia and tarsus of leg IV (right, antiaxial aspect) **J** Femur and genu of leg IV (right, antiaxial aspect).

*Legs* (Fig. 2): Lateral claws thinner than middle one, having small, but distinct serrations on dorsal edge (Fig. 2G). Setation of legs typical for genus, most setae finely barbed except few distal or ventral setae on tarsi, femora and trochanters. Solenidia φ_1_ on tibiae I about 2.8 times as long as φ_2_; setae *l*” on genua I and II markedly thick; setae *s* on tarsi II very thick, bearing several strong branches; genua IV curved, markedly longer than others. Formula of setation, including famuli: I (1-5-3-4-20), II (1-5-3-4-15), III (2-2-1-3-15), IV (1-2-2-3-12); formula of solenidia: I (1-2-2), II (1-1-2), III (1-1-0), IV (0-1-0); homology of setae and solenidia as indicated in Table 1.

**Table 1.** Homology of leg setation and solenidia of Achipteria (Izuachipteria) setulosa (Golosova, 1981)*

Legs	Trochanter	Femur	Genu	Tibia	Tarsus
I	*v*’	*d*, (*l*), *bv*”, *v*”	(*l*), *v*’, σ	(*l*), (*v*), φ_1_, φ_2_	(*ft*), (*tc*), (*it*), (*p*), (*u*), (*a*), *s*, (*pv*), *v*’, (*pl*), *l*”, *e*, ω_1_, ω_2_
II	*v*’	*d*, (*l*), *bv*”, *v*”	(*l*), *v*’, σ	(*l*), (*v*), φ	(*ft*), (*tc*), (*it*), (*p*), (*u*), (*a*), *s*, (*pv*), ω_1_, ω_2_
III	*l*’, *v*’	*d*, *ev*’	*l*’, σ	*l*’, (*v*), φ	(*ft*), (*tc*), (*it*), (*p*), (*u*), (*a*), *s*, (*pv*)
IV	*v*’	*d*, *ev*’	*d*, *l*’	*l*’, (*v*), φ	*ft*”, (*tc*), (*p*), (*u*), (*a*), *s*, (*pv*)

* Roman letters refer to normal setae, *e* to famulus; Greek letters to solenidia; single prime (’) marks setae on anterior and double prime (”) setae on posterior side of the given leg segment; parentheses refer to a pair of setae.

#### Remarks.

The character states of the specimens examined here accord well with those studied by Golosova (1981). Only the slight differences are the scarcely barbed sensilli in the Russian specimens (smooth in Japanese specimens), and number of epimeral setae (Russian specimens has fewer setae than Japanese ones). Until now, the present species was known only from the type locality, Kuril Islands in the Russian Far East. The original description, illustration and differential diagnosis of this species were not sufficient, and hence we present here some supplementary details.

Achipteria (Izuachipteria) setulosa resembles the two other Japanese species, Achipteria (Izuachipteria) alpestris and Achipteria (Izuachipteria) imperfecta in having short and slender interlamellar setae. However, Achipteria (Izuachipteria) alpestris is different from Achipteria (Izuachipteria) setulosa by the strongly-developed median horn-like projection of the rostrum, the relatively shorter sensilli, and much smaller body size. Another Japanese species, Achipteria (Izuachipteria) imperfecta has no interlamellar setae, relatively thick sensilli, different dentation of lamellar cusps, and much smaller body size.

## Discussion

In the comprehensive checklist of oribatid mites of Japan, Fujikawa et al. (1993) presented eight species of Achipteriidae belonging to five genera, namely *Achipteria*, *Anachipteria*, *Parachipteria*, *Hokkachipteria* and *Izuachipteria*, but the two latter taxa are now considered as subgenera of *Achipteria*.

Most of achipteriid species found in Japan are known to be widely distributed in vast areas of the northern hemisphere. Thus, *Achipteria coleoptrata* (Linnaeus, 1758), *Achipteria curta* Aoki, 1970, *Achipteria nitens* (Nicolet, 1855), *Anachipteria achipteroides* (Ewing, 1913) and *Parachipteria punctata* (Nicolet, 1855) are widely distributed through Holarctic region. Some of these species were also recorded from the other biogeographic regions, e.g. in addition to their common distributions in Europe (everywhere), North America (USA and Canada), and Asia (Russian Far East, Siberia, Kazakhstan, Mongolia and Japan), *Achipteria coleoptrata*, *Achipteria curta* and *Parachipteria punctata* were reported from India, Vietnam, subtropical part of China and Santa Helena islands (Wallwork 1977, Haq and Sumangala 2003, Wang et al. 2003, Chen et al. 2010). Two other species, such as *Anachipteria grandis* Aoki, 1966 and *Parachipteria distincta* (Aoki, 1959) have also fairly wide distributions in the Palaearctic region. Only four species, Achipteria (Izuachipteria) alpestris, Achipteria (Izuachipteria) imperfecta, Achipteria (Izuachipteria) setulosa and *Parachipteria truncata* Aoki, 1970 have restricted distributions mainly in Japan, but two of these, (Achipteria (Izuachipteria) imperfecta and Achipteria (Izuachipteria) setulosa) have extended distributions in Taiwan and the Russian Far East (Aoki 1991, Ryabinin and Pan’kov 2002, Ohkubo et al. 2015, Subías 2015).

Among these species, *Parachipteria distincta* is most common species in Japan, which is ubiquitous in this country. Some other species, such as *Achipteria curta*, Achipteria (Izuachipteria) alpestris, Achipteria (Izuachipteria) imperfecta and *Anachipteria grandis* are rather common, especially in its northern and central regions of the country. The other species (*Achipteria coleoptrata*, *Achipteria nitens*, *Achipteria serrata*, *Anachipteria achipteroides*, *Parachipteria truncata*, *Parachipteria punctata*) are relatively rare, and known to be distributed only in one prefecture each. Most species of Achipteriidae in Japan are the inhabitants of the litter of various forests, such as natural broad leaved forests in high mountainous areas, soils of grasslands, wetlands and mosses growing on rocks.

As mentioned above, *Achipteria* *sensu lato* is the largest genus of Achipteriidae, and it encompasses diverse species in terms of morphological characters. Balogh and Mahunka (1979) attempted to classify species of *Achipteria* using the size of the interlamellar setae, but this proposal was not broadly accepted. In this sense, validity of the subgenus Achipteria (Izuachipteria) might not acceptable, but further detailed studies are required on the morphology of both adults and immature stages to clarify the status of this subgenus, which is beyond the scope of the present work.

The structure of lamellar complex is quite diverse in various species of *Achipteria*, e.g. some species have anteriorly narrowed, elongate triangular lamellae pointed distally with sharp lateral cusps, which is a typical lamellar complex for Achipteriidae and an apomorphic character, according to Weigmann (2010). The other species have very broad lamellae distally with large cusps, which is a plesiomorphic character, according to the above-mentioned author. In case of the latter lamellar complex, the distal ends of lamellar cusps are mostly bent downwards, but in various species, these bending cusps are being either dentate or evenly rounded distally. Based on these different characters, it might be possible to establish at least two subgenera within the genus *Achipteria*. However, we do not do so, because of the below given reason.

As stated by Weigmann (2010) there are many genera of oribatid mites, creation of which were based upon single conspicuous character or some combination of characters, whose value for assessing phylogenetic relations is questionable. Moreover, Behan-Pelletier (2001) and Lindo et al. (2008) declared that the shapes of the lamellae vary extensively not only within the family Achipteriidae, but even among different families of poronotic Brachypylina, and the polarity of these variations is unclear. They justified that proposing a separate generic taxon based on character of the lamellae is not appropriate.

Although it is not preferable to establish new subgeneric level taxa based on the characters of lamellar complex, it is suggested to classify the known species of *Achipteria* into three species-groups. The first species-group, which we call the *coleoptrata*-group, has lamellar complex with anteriorly narrowed, elongate triangular lamellae pointed distally with sharp lateral cusps, but without medial cusps. Besides the type species, *Achipteria coleoptrata*, this species-group includes such species as *Achipteria bicarinata* Moskacheva, 1973, *Achipteria borealis* (Banks, 1889), *Achipteria cucullata* Moskacheva, 1973, *Achipteria elegans* Schweizer, 1956, *Achipteria holomonensis* Cancela da Fonseca & Stamou, 1987, *Achipteria italica* (Oudemans, 1914), *Achipteria oregonensis* Ewing, 1918, *Achipteria quadridentata* (Willmann, 1951) and *Achipteria sumatrensis* Willmann, 1931.

The second species-group, the *serrata*-group, has very broad lamellar complex, and the cusps are distally serrated with various dens or teeth. *Achipteria serrata* has strong serration on the distal end of lamellar cusps, and some other species with same character could be included in this group, e.g. Achipteria (Izuachipteria) alpestris, *Achipteria curta*, Achipteria (Izuachipteria) setulosa and *Achipteria catskllensis* Nevin, 1977.

The third group, the *nitens*-group, has similar structures of the lamellar complex to the *serrata*-group, but the distal end of lamellar cusps are not serrated, i.e. bluntly rounded or sometimes with pointed lateral tooth. This species-group includes *Achipteria baleensis* Ermilov, Rybalov & Kemal, 2011, *Achipteria clarencei* Nevin, 1977, *Achipteria hasticeps* (Hull, 1914), Achipteria (Izuachipteria) imperfecta, *Achipteria nitens* (Nicolet, 1855), *Achipteria longesensillus* Schweizer, 1956, *Achipteria longisetosa* Weigmann & Murvanidze, 2003 and *Achipteria verrucosa* Rjabinin, 1974.

This grouping might be useful for further classification of *Achipteria* species, and it should mentioned here that we do not include some hitherto known species of *Achipteria* (e.g. *Achipteria armata* (Banks, 1895), *Achipteria hasticeps* (Hull, 1914), *Achipteria languida* (Nicolet, 1855), *Achipteria minuta* (Ewing, 1909), *Achipteria moderatior* Berlese, 1923 etc.) into any species-group, due to their unclear diagnostic characters.

It is evident that the large lamellar complex is for protecting the dorsal, lateral and anterior parts of the prodorsum and especially the anterior legs in redrawn position, but in some species of *Achipteria* the lamellar complex became distinctly smaller; the structure and function of different lamellar complexes are the interesting topics of the future studies.

In conclusion, the following key can be used to identify the adults of all known species of Achipteriidae in Japan.

### A key to adults of known species of Achipteriidae in Japan

**Table:** 

1	Octotaxic system expressed as four pairs of notogastral porose areas	**2**
–	Octotaxic system expressed as four pairs of notogastral saccules instead of poros areas (*Achipteria* *sensu lato*)	**3**
2	A knife-like humeral projection of pteromorphs lacking (*Anachipteria*)	**9**
–	Pteromorphs with a knife-like humeral projection (*Parachipteria*)	**10**
3	Lamellar and interlamellar setae long, setae *le* extending beyond anterior tip of lamellar cusps; setae *in* not extending far beyond basis of lamellar cusps (Achipteria (Achipteria))	**4**
–	Lamellar and interlamellar setae short, thin, sometimes setae *in* absent; setae *le* not reaching anterior tip of lamellar cusps; setae *in* not reaching basis of lamellar cusps Achipteria (Izuachipteria)	**7**
4	Lamellar cusps rounded or with large lateral dens; sensilli long	**5**
–	Lamellar cusps without lateral dens, but medially with 3-4 small dens; sensilli short	**Achipteria (Achipteria) curta Aoki, 1970**
5	Notogastral setae well developed; lamellar cusps broad distally, concave medially, with few serrations or rounded distally	**6**
–	Notogastral setae minute or represented by their alveoli; lamellar cusps with large, elongate-triangular lateral dens	**Achipteria (Achipteria) coleoptrata (Linnaeus, 1758)**
6	Notogastral setae long, especially setae *c* and *la* very long; lamellar cusps with small lateral dens or blunt at tip; interlamellar setae extending beyond lamellar cusps; sensilli long, slender	**Achipteria (Achipteria) nitens (Nicolet, 1855)**
–	Notogastral setae *c* and *la* medium long, other setae very short; lamellar cusps with large lateral dens, concave medially and with few serrations; interlamellar setae not reaching tip of lamellar cusps; sensilli short, club-shaped	**Achipteria (Achipteria) serrata Hirauchi & Aoki, 1997**
7	Interlamellar setae short, thin, but conspicuously developed; anterior margin of lamellar cusps distinctly serrated	**8**
–	Interlamellar setae absent; anterior margin of lamellar cusps not serrated, but bluntly rounded	**Achipteria (Izuachipteria) imperfecta (Suzuki, 1972)**
8	Rostrum with strongly-developed median horn-like projection; lamellar setae long, thick, reaching anterior end of cusps; body size relatively small (550-610 μm)	**Achipteria (Izuachipteria) alpestris (Aoki, 1973)**
–	Rostrum rounded, without median horn-like projection; lamellar setae short, thin, not reaching anterior end of cusps; body size large (718–796 μm)	**Achipteria (Izuachipteria) setulosa (Golosova, 1981)**
9	Sensilli fusiform, long, extending far anterior to pedotecta I; lamellar cusps without medial dens; lamellar setae smooth	***Anachipteria achipteroides* (Ewing, 1913)**
–	Sensilli club-shaped, short, not reaching level of the anterior end of pedotecta I; lamellar cusps with distinct medial dens; lamellar setae barbed	***Anachipteria grandis* Aoki, 1966**
10	Relatively small species with body length less than 450 μm; notogastral porose areas large; notogaster without granular punctuations	**11**
–	Relatively large species with body length greater than 550 μm; notogastral porose areas small; notogaster with large granular punctuations	***Parachipteria punctata* (Nicolet, 1855)**
11	Lamellar cusps with blunt, but distinct medial dens, lateral dens large; region between medial and lateral dens of lamellar cusps deeply concaved; interlamellar setae extending beyond anterior end of lamellae	***Parachipteria distincta* (Aoki, 1959)**
–	Lamellar cusps truncate, without medial dens; end of lamellar cusps not concaved, but convex, with few small teeth; interlamellar setae not reaching anterior end of lamellae	***Parachipteria truncata* Aoki, 1976**

## Supplementary Material

XML Treatment for Achipteria (Izuachipteria) setulosa
